# Retention and loss of PIT tags and surgically implanted devices in the Eurasian beaver

**DOI:** 10.1186/s12917-022-03333-1

**Published:** 2022-06-10

**Authors:** Martin Mayer, Marianne Lian, Boris Fuchs, Christian A. Robstad, Alina L. Evans, Kathryn L. Perrin, Eva M. Greunz, Timothy G. Laske, Jon M. Arnemo, Frank Rosell

**Affiliations:** 1 Department of Natural Sciences and Environmental Health, University of South-Eastern Norway, Bø i Telemark, Norway; 2 Department of Ecoscience, Aarhus University, Grenåvej 14, 8410 Rønde, Denmark; 3 Department of Forestry and Wildlife Management, Inland Norway University of Applied Sciences, Koppang, Norway; 4 Center for Zoo and Wild Animal Health, Copenhagen Zoo, Frederiksberg, Denmark; 5 San Diego Zoo Wildlife Alliance, San Diego Zoo Safari Park, Escondido, CA USA; 6 Department of Surgery, University of Minnesota, Minneapolis, USA; 7 Department of Wildlife, Fish and Environmental Studies, Swedish University of Agricultural Sciences, Umeå, Sweden

**Keywords:** Animal welfare, Body temperature logger, *Castor fiber*, Heart rate logger, Surgery

## Abstract

**Background:**

Passive integrated transponder devices (PIT tags) are a valuable tool for individual identification of animals. Similarly, the surgical implantation of transmitters and bio-loggers can provide useful data on animal location, physiology and behavior. However, to avoid unnecessary recapture and related stress of study animals, PIT tags and bio-loggers should function reliably for long periods of time. Here, we evaluated the retention of PIT tags, and of very high frequency (VHF) transmitters and bio-loggers that were either implanted subcutaneously or into the peritoneal cavity of Eurasian beavers (*Castor fiber*).

**Results:**

Over a 21-year period, we implanted PIT tags in 456 individuals and failed to detect a PIT tag at recapture in 30 cases, consisting of 26 individuals (6% of individuals). In all instances, we were still able to identify the individual due to the presence of unique ear tag numbers and tail scars. Moreover, we implanted 6 VHFs, 36 body temperature loggers and 21 heart rate loggers in 28 individuals, and experienced frequent loss of temperature loggers (at least 6 of 23 recaptured beavers) and heart rate loggers (10 of 18 recaptured beavers). No VHFs were lost in 2 recaptured beavers.

**Conclusions:**

Possible causes for PIT tag loss (or non-detection) were incorrect implantation, migration of the tag within the body, a foreign body reaction leading to ejection, or malfunctioning of the tag. We speculate that logger loss was related to a foreign body reaction, and that loggers were either rejected through the incision wound or, in the case of temperature loggers, possibly adhered and encapsulated to intestines, and then engulfed by the gastro-intestinal tract and ejected. We discuss animal welfare implications and give recommendations for future studies implanting bio-loggers into wildlife.

**Supplementary Information:**

The online version contains supplementary material available at 10.1186/s12917-022-03333-1.

## Background

Passive integrated transponder devices (PIT tags; also known as ‘microchips’) are valuable for studying animal ecology and behavior, because they enable individual identification of animals [1, 2]. They are widely used due to their small size and comparatively low price, but do not allow to actively track animals, thus only providing data when the PIT tag is scanned (usually upon recapture or via scanners installed at fixed locations). To obtain more detailed relocation data and allow for active tracking of animals, very high frequency (VHF) transmitters and global positioning systems (GPS) are often used [3, 4]. These devices usually are larger and more expensive than PIT tags, with attachment methods varying depending on the studied species. In cases where external attachment is not possible, e.g. via collars or backpacks, VHFs can be implanted surgically. Similarly, the surgical implantation of temperature and heart rate loggers can provide highly useful data on physiological parameters and behavior [5–9]. This is especially true for cryptic species that are hard to monitor and observe directly, such as nocturnal animals.

Intra-peritoneal bio-loggers are increasingly used in physiological field research worldwide [9–11]. Several mammal studies have used either subcutaneous or intra-peritoneal loggers [5, 12–14]. Most studies have reported successful results without complications, whereas others document varying tissue responses. A study on brown bears (*Ursus arctos*) reported corrosion of parts of implanted devices and leakage from short-circuited batteries, which resulted in the death of two study animals [13]. The same study found a varying degree of tissue foreign body reaction to the implanted devices, ranging from no reaction to light encapsulation to thick capsule formation.

Additionally, the implantation process and associated anesthesia can cause complications, e.g. due to circulatory failure [15], and more generally the capture process can be highly stressful for the studied animals [16–18]. Thus, to avoid unnecessary recapture of study animals, PIT tags and bio-loggers should function for long periods of time, and their functionality and retention should be tested. For example, a study on American black bears (*Ursus americanus*) found that 5 of 6, including 2 of 2 intraperitoneal and 3 of 4 subcutaneous devices, were rejected, potentially due to foreign body responses [19]. Similarly, 25 of 39 subcutaneous implants were rejected prematurely between 2 and 198 days post-implantation in American black bear cubs [20].

Here, we evaluated the retention of PIT tags, implanted VHF transmitters, and bio-loggers that were either implanted subcutaneously or into the peritoneal cavity of Eurasian beavers (*Castor fiber*; hereafter beaver). Beavers are large semiaquatic rodents. They are ecosystem engineers that can facilitate aquatic and semi-aquatic biodiversity and can be used for biodiversity conservation [21, 22], making them an important wildlife species to study. Visual identification of unmarked individuals is usually impossible (apart from tail scar identification), because they are sexually monomorphic and nocturnal. Thus, apart from ear tagging [23, 24], PIT tagging is a common technique to mark and identify individuals [25, 26], and might allow for remote monitoring of beavers [27]. Moreover, the implantation of VHF transmitters was previously used to study the movement of beavers [15]. However, to our knowledge, this is the first study describing the implantation and retention of body temperature loggers and insertable cardiac monitors (measuring heart rate) in beavers.

## Methods

### Study areas, beaver captures, and PIT tagging

We captured beavers in two study areas in Norway, located around Bø, Vestfold and Telemark County (59.38˚N, 9.17˚E) and around Evenstad, Innlandet County (61.42˚N, 11.09˚E), with permission from the relevant authorities and the local landowners (see ethics approval). The first study area consisted of three medium-sized rivers (for details see [28]), and the second area of the large river Glomma. Only the first area was used for PIT tag evaluation, because this was our long-term study area where we have studied beavers since 1997 (beavers in Evenstad were only recaptured to retrieve temperature and heart rate loggers; see below). Beavers were captured from a motorboat at night, using spotlights and landing nets [29]. From 1997–2017, we captured 456 individuals between 1 and 24 times (mean ± SD: 3.1 ± 3.3; median: 2), totaling 1,391 capture events. We removed captures of the same individual within the same year to avoid pseudoreplication, leaving 1,027 capture events.

All newly captured individuals were implanted with a PIT tag (iTag162, BTS-ID, Helsingborg, Sweden) that was implanted subcutaneously in the dorsal midline neck region with a specialized needle (NE-100/162, BTS-ID) and implanter (IMP-90pit, BTS-ID), and with unique ear-tags [30]. All PIT tags were checked for functionality upon implantation. We recorded tail scars using a field sheet. Individuals were sexed based on the color and viscosity of their anal gland secretion [31]. The age of individuals first captured as kit or yearling was determined based on body mass [32], and we classified beavers first captured as adults, as minimum two years old when they had a mass ≥ 17 kg and ≤ 19.5 kg, or as minimum three years when > 19.5 kg at the time of first capture [30]. We categorized beavers into kits (first year of life), yearlings (second year of life), 2–3 year old, and ≥ 4 year old, because growth rates differ between these age classes [33]. Upon each recapture, we used a scanner (MiniTracker 3, Avid Identification Systems, Inc., Norco, USA) to detect the PIT tag. The PIT tag was considered malfunctioning or lost if not detectable after we scanned the entire body of the beaver.

### Implantation and retrieval of bio-loggers

In August 2015 and October 2016 we implanted 36 body temperature loggers in 28 beavers (8 individuals received a second temperature logger after the first one was removed/not recovered), with 18 of these individuals also receiving 21 cardiac monitors (hereafter heart rate loggers; 3 individuals received two heart rate loggers; Table 1, Table S1 and Table S2). We used two temperature logger models (*DST centi-T*, ceramic and epoxy coating, diameter × length: 15 × 46 mm, weight: 19 g or *DST micro-T*, 8.3 × 25.4 mm, 3.3 g; both produced by Star-Oddi, Garðabær, Iceland), and three heart rate logger models (Reveal DX and XT, both having the same coating of titanium, silicone, and parylene, dimensions of both: 8 mm × 19 mm × 62 mm, 15 g; and Reveal LINQ, titanium coating, 4.0 mm × 7.2 mm × 44.8 mm; 2.4 g; all Medtronic Inc., Minneapolis, Minnesota, USA). In the Evenstad population, no prior marked (ear-tagged or PIT-tagged) individuals were available, and we implanted free floating VHF transmitters (model 1245B, diameter × length: 20 mm × 70 mm, weight: 40 g, Advanced Telemetry Systems, Isanti, Minnesota, USA) into the peritoneal cavity to facilitate recapture.

**Table 1:** Overview of the number of temperature loggers and heart rate loggers implanted in Eurasian beavers (*Castor fiber*) shown separately by sensor type, sex and fate

Sensor type/sex	Not recaptured	Lost/undetected	Recovered	Total
*Temperature loggers*				
**Centi-T**	**10**	**5**	**11**	**26**
Female	4	2	5	11
Male	6	3	6	15
**Micro-T**	**3**	**4**	**3**	**10**
Female	2	1	2	5
Male	1	3	1	5
**Total**	**13**	**9**	**14**	**36**
*Heart rate loggers*				
**RVL-BW**	**2**	**9**	**3**	**14**
Female		4	2	6
Male	2	5	1	8
**RVL-XT**			**3**	**3**
Female			2	2
Male			1	1
**Linq**	**1**	**1**	**2**	**4**
Female		1		1
Male	1		2	3
**Total**	**3**	**10**	**8**	**21**

After capture, beavers were transported in cloth sacks to the veterinary processing area. Anesthesia was induced with intramuscular medetomidine (0.05 mg/kg, Domitor vet 1 mg/ml, Orion Pharma), butorphanol (0.1 mg/kg, Butomidor® vet 10 mg/ml, Richter Pharma), ketamine (5 mg/kg, Narketan® 100 mg/ml, Vetoquinol), and midazolam (0.24 mg/kg Midazolam B Braun 5 mg/ml). After induction of anesthesia, all beavers received meloxicam (0.2 mg/kg Metacam 5 mg/ml, Boehringer Ingelheim), and intra-nasal oxygen 0.5 L/min; via oxygen bottle or via an oxygen concentrator (setting 4.5 – 6, Eclipse 5™ auto SAT ® SeQual, Medtek.no). For surgery, individuals were put in dorsal recumbency. An area caudal to the umbilicus (approx. 6 × 4 cm) was clipped and surgically prepared with chlorhexidine in 60% ethyl alcohol (Klorhexidinsprit 5 mg/ml Fresenius Kabi). For access to the peritoneal cavity, an approximately 4 cm long ventral midline incision through the *Linea alba* was made using standard surgical techniques. Temperature loggers and VHF transmitters were gas sterilized using ethyline oxide gas. VHF transmitters and temperature loggers were placed in the peritoneal cavity and sutured to the ventral body wall at the level of the umbilicus using non-absorbable monofilament suture material (Prolene™, Ethicon). The incision was closed in two–three layers (*Linea alba*, subcutaneous tissue when sufficient, and skin) with absorbable monofilament suture material (PDS™ II, Ethicon), using a simple interrupted pattern for the *Linea alba* (US 0; with a round needle), a simple continuous pattern using the same suture for the subcutaneous layer, and finally an intradermal or interrupted horizontal mattress pattern for the skin (US 2–0, cutting needle). The skin incision was covered with a spray dressing (OPSITE, Smith & Nephew Medical Ltd). Heart rate loggers were inserted through a 1–2 cm incision (depending on device size) subcutaneously on the left lateral thorax at the level of the heart. The skin was closed with intra-dermal sutures (PDS US 2–0). Surgeries for implanting both loggers lasted for 21 ± 6 min (mean ± SD).

**Fig. 1:**
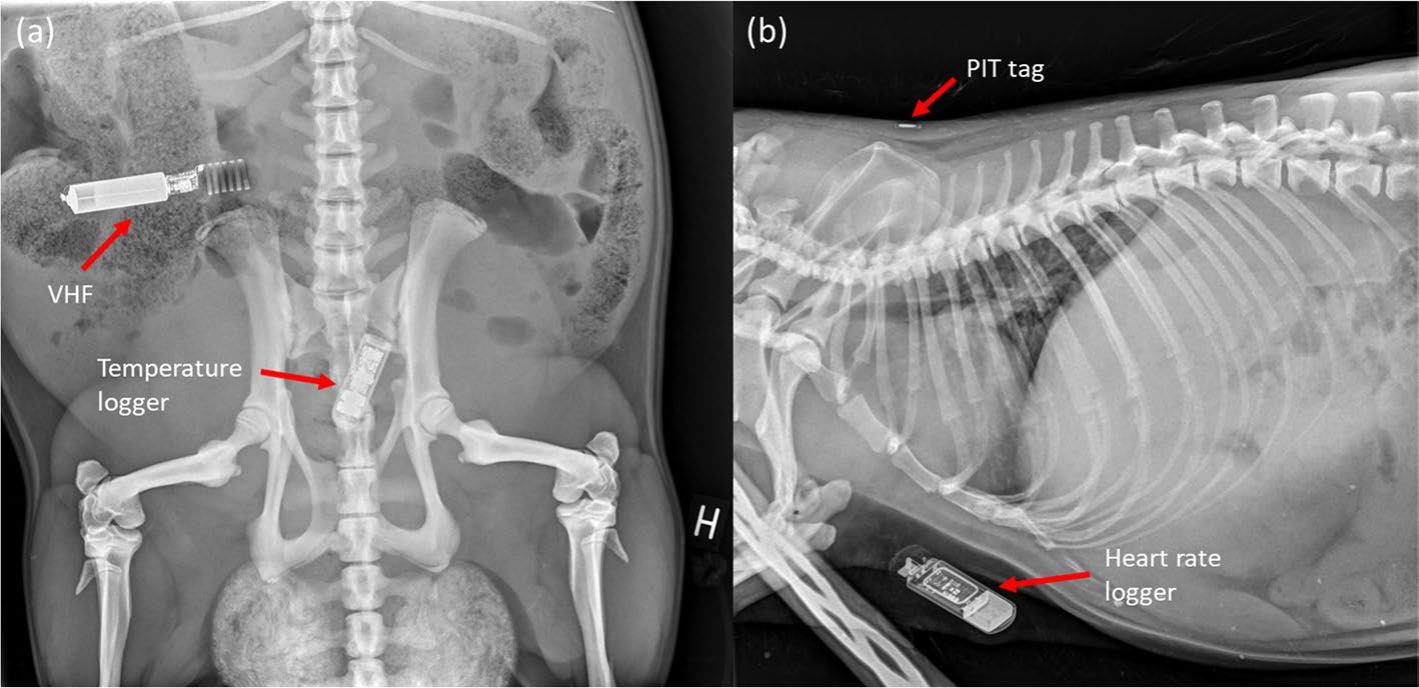
Dorsoventral and right lateral radiographs of two Eurasian beavers (*Castor fiber*) demonstrating the location of (**a**) a temperature logger (center) and VHF (top left) within the peritoneal cavity, and (**b**) a subcutaneous heart rate logger (bottom) and a subcutaneous PIT tag (top)

Retrieving loggers at recapture followed the same procedure, except surgery times lasted 37 ± 19 min and abdominal incision lengths were approximately 2 cm longer to facilitate removal. After surgery ended, anesthesia was partially antagonized with intramuscular atipamezole (0.25 mg/kg, Antisedan 5 mg/ml, Orion Pharma). Beavers were released close to the main lodge of their own territory once they were fully recovered, typically 4 h post-surgery. Approximately six months to one-year post-surgery, we attempted to recapture the beavers to download heart rate logger data and to remove the temperature loggers. The downloading of heart rate logger data was performed using a transcutaneous telemetry system with beavers restrained in a cloth sack without anesthesia [34]. Temperature loggers had to be removed from the peritoneal cavity to access data. The anesthesia and removal of temperature loggers and VHF transmitters were conducted simultaneously. After 3 surgeries where loggers were not found, the remaining beavers were radiographed before the logger removal surgery to confirm if loggers were present (Fig. 1). When radiography showed that loggers were no longer present in a beaver’s body, we did not conduct surgery.

One of the beavers died during anesthesia prior to surgery for logger retrieval. The postmortem examination revealed severe cardiac compromise due to moderately dilated cardiomyopathy and myocardial fibrosis, most likely leading to death due to cardiac failure during induction. Necropsy revealed that the body temperature logger and VHF implant were present, and the heart rate logger was lost. To the best of our knowledge, no other beaver in this study was injured during capture and handling.

### Statistical analyses

We analyzed the probability of PIT tag loss in a given year (1 = lost versus 0 = not lost; response variable; *n* = 1,027 capture events of 456 individuals) using a generalized linear mixed model of the R package ‘lme4’ [35] with a binomial distribution, including, age class (yearling, 2–3 year old, > 3 year old) and sex as fixed effects and beaver ID and year as random intercept. Kits were excluded from this analysis, because they never lost a PIT tag (see Results). For temperature (*n* = 36) and heart rate loggers (*n* = 21), we also analyzed the probability of logger loss (two separate analyses) using a generalized linear model with a binomial distribution. We included sex and the sensor type as independent variables. We included sensor type to test the hypothesis that the size of the logger (which differed between models) affects the probability of logger retention. We did not include age, because all individuals that received a temperature or heart rate logger were adults (≥ 2 years old), and in order to avoid overfitting our statistical models due to the small sample size. For all analyses, we selected the most parsimonious model based on AIC, by comparing the full model, single effects models, and the intercept only model using the R package ‘MuMIn’ ([36] Table 2). If two or more models had AIC values within ∆AIC < 2, we selected the simpler model [37]. Validation of the most parsimonious model was made by visual inspection of residuals [38]. Model estimates that included zero within their 95% confidence interval were considered uninformative [39]. All statistical analyses were carried out in R 4.0.3 [40].

**Table 2:** The model selection results for the analyses investigating the probability of (1) PIT tag loss, (2) temperature logger loss, and (3) heart rate logger loss, showing the used degrees of freedom (df), log likelihood (logLik), AIC_c_, ∆AIC, and AIC weight. Models were ranked by AIC_c_

Model	df	logLik	AICc	∆AIC	AIC weight
(1) PIT tag loss					
Intercept only	2	-114	231	0.00	0.484
Sex	3	-113	232	1.05	0.286
Age class	4	-113	234	2.44	0.143
Age class + Sex	5	-112	235	3.44	0.087
(2) Temperature logger loss					
Intercept only	1	-15	33	0.00	0.451
Sensor type	2	-15	34	1.06	0.266
Sex	2	-15	35	1.78	0.185
Sensor type + Sex	3	-14	36	3.07	0.097
(3) Heart rate monitor loss					
Intercept only	1	-12	27	0.00	0.564
Sensor type	2	-12	29	1.83	0.226
Sex	2	-12	30	2.55	0.157
Sensor type + Sex	3	-12	32	4.73	0.053

## Results

### PIT tags

**Fig. 2:**
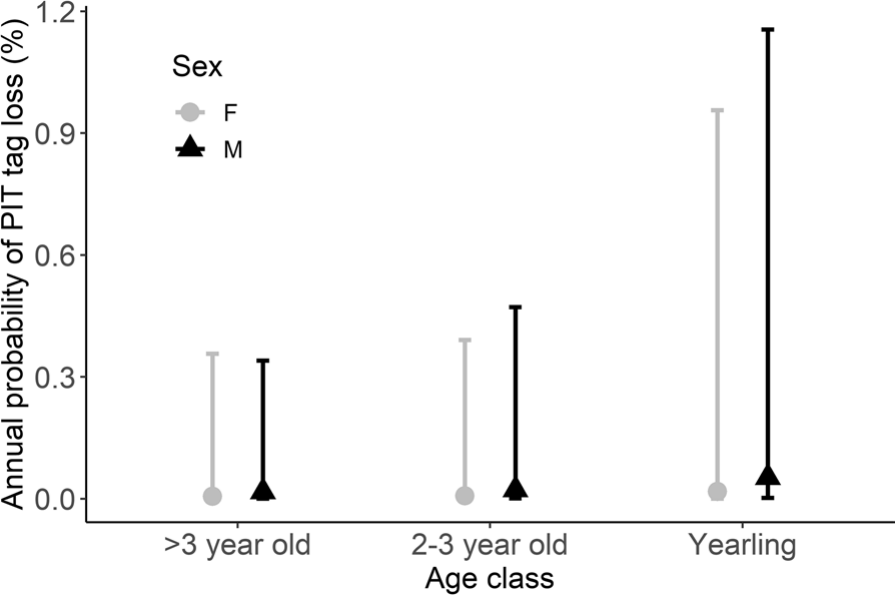
The annual probability of PIT tag loss in Eurasian beavers (*Castor fiber*), separately by age class and sex (grey dots = female, black triangles = male). Bars show 95% confidence intervals. Note that these effects were uninformative

Of the 1,027 capture events (456 individuals), we failed to detect a PIT tag in 30 cases (2.9% of capture events and 6.6% of all individuals). Consequently, we implanted a new PIT tag in these individuals, i.e., 26 individuals ‘lost’ their PIT tag 1–3 times (mean ± SD: 1.2 ± 0.5). In all instances we were still able to identify the individual due to the presence of unique ear tag numbers and tail scars. We implanted PIT tags in 105 kits captured for the first time, and always detected the PIT tag when recapturing these individuals within their first year of life (51 recaptures). We failed to detect PIT tags in 10 individuals out of 87 yearlings (11.5%), 10 cases (8 individuals) out of 190 2–3 year olds (5.3%), and 10 cases (8 individuals) out of 74 ≥ 4 year old individuals (13.5%). When calculating the rate of PIT tag disappearance per beaver year, PIT tag disappearance was 0.0% in kits, 7.0% in yearlings (i.e., in the year from being a kit to being a yearling), 2.9% in 2–3 year olds, and 2.6% in ≥ 4 year olds. Of the 30 PIT tag disappearances, 8 were in females (5 in yearlings, 1 in 2–3 year olds, and 2 in ≥ 4 year olds) compared to 22 in males (5 in yearlings, 9 in 2–3 year olds, and 8 in ≥ 4 year olds). The overall sex ratio in our study population was even (223 females, 233 males). The percentage of PIT tag disappearance also varied among years, from 0 to 9.5% (mean ± SD: 3.0 ± 2.9%), being > 5% in 6 out of 21 years. The annual probability of PIT tag loss was best explained by the intercept only model (Table 2).When considering the full model, annual probability of PIT tag loss was not related to the age class or sex (Fig. 2, Table S3).

### Temperature loggers, heart rate loggers and VHFs

Of the 36 temperature loggers that we implanted into beavers (Table 1, Table S1), we subsequently retrieved 13 loggers by recapturing individuals and one from a beaver that was shot by a hunter. The presence of the temperature logger was confirmed by using radiography in 5 individuals (we did not have access to radiography equipment during the other recaptures). In addition, in 9 recaptured beavers, we failed to recover the temperature logger, and the loss of loggers was confirmed by radiography in 6 cases (radiography equipment was not available in the other recaptures). Finally, 13 loggers were not retrieved, because we failed to recapture the beaver (Table 1). The probability of temperature logger loss (in the 23 cases when we recaptured beavers) was best explained by the intercept only model (Table 2), and logger type and sex were uninformative in explaining logger loss in the full model (Table S4).

Of the 21 beavers with heart rate loggers, we could recapture 18 individuals between 1 and 6 times, and downloaded data from 8 heart rate loggers (Table 1, Table S2). For 10 individuals, we could confirm that they had lost the heart rate logger; for 3 of these individuals we were able to confirm the presence of the logger 3–6 months post-implantation, prior to subsequent logger loss (Table S2). Due to the subcutaneous location of the heart rate logger we were able to confirm its presence or absence by palpation. Three beavers could not be located and recaptured (Table 1, Table S2). The probability of heart rate logger loss (in the 18 cases when we recaptured beavers) was best explained by the model including the sensor type, but this model was within ∆AIC < 2 of the intercept only model (Table 2). Moreover, sensor type and sex were uninformative in explaining logger loss in the full model (Table S4) and in the model including sensor type.

Of the 6 beavers with VHF units implanted, we recaptured 2 and retrieved their VHF units (no other individuals with an implanted VHF were recaptured). Expected battery life was 540 days (at 40 beats per minutes and 20 ms signal length) but neither unit pulsed a signal 381 days after implantation. The VHF units were regularly monitored post implantation for up to 192 days when ice covered the river in mid-November. Signal strength was weak when beavers were below ground and disappeared with the ice and snow cover. No signals were present one-year post implantation either due to unit failure, empty battery or due to the beavers leaving the study area.

## Discussion

### PIT tags

The overall rate of PIT tag retention in our study was high, as shown for other species [41], emphasizing that they are a useful and reliable tool to mark individuals. Additional marking methods, such as ear tags or characteristic body markings/coloration [23, 42], can be used as a security mechanism to ensure accurate identification of individuals. Individual marking with a PIT tag or ear tags alone would have, in some instances, resulted in our inability to identify individuals after some years.

The analysis investigating the probability of PIT tag loss was best explained by the intercept only model. Nevertheless, the raw data on annual PIT tag loss suggests that PIT tags were ca. 2-times more often rejected in the year between the first capture as a kit and the next capture as a yearling, compared to older individuals. Our personal experience is that kits were harder to implant with PIT tags compared to older individuals, potentially because the skin is less elastic with a smaller subcutaneous space for PIT tag placement. Thus, we speculate that the main cause for PIT tag disappearance was incorrect implantation technique, with PIT tags positioned too close to the skin entry site, and subsequently ejected. This could also explain the variation in the rate of PIT tag loss among years, i.e., when new personnel with less experience joined the project. An alternative explanation could be the large growth from kit to yearling, compared to older individuals. This might have either led to the ejection of the PIT tag or the movement of the PIT tag to another position in the body, as shown in other species [43], consequently not being detected by us. However, we scanned the entire beaver body when we did not detect the PIT tag in the neck, but failed to detect PIT tags in other body regions. We cannot exclude the possibility that failure to detect PIT tags, especially in older individuals, might be caused by the failure of the PIT tag [44].

### Temperature loggers, heart rate loggers, and VHF

In the 23 instances where we recaptured beavers, 14 temperature loggers were retrieved from the peritoneal cavity, while 9 loggers were not found, in spite of radiographing beavers in 6 of these cases. This suggests that the logger had been lost by the beaver. Similarly, the fact that ca. half of all beavers lost their heart rate logger suggests that the logger or the suture material caused a foreign body reaction by the beavers’ immune system, resulting in loss of the heart rate logger. Implant loss was also reported in American black bears, where both subcutaneous and intraperitoneal bio-loggers were externalized > 44 days post-implantation [19], with fewer rejections occurring in subsequent studies that used the smaller ‘Reveal LINQ’ heart rate model [45]. We found no differences among models (that markedly differed in size), which does not support the idea that smaller loggers are more likely to be rejected. However, we only implanted 3 ‘LINQ’ heart rate loggers, a sample size too small to make a reliable comparison between heart rate logger models. For the VHF, two animals were recaptured, and both had the VHF in place.

Most likely, the loss of subcutaneous heart rate loggers was due to a foreign body reaction to either the logger or the suture material used. Suture material takes 180–210 days for complete absorption [46], which spans most of the timespan for observed logger loss. One beaver had edema around the spot where the logger had previously been at recapture (> 6 months after implantation), indicating recent loss and making a foreign body reaction likely. The fact that beavers were swimming shortly after recovery, before the surgical wound was healed, could be a contributing factor. However, other aquatic species have received surgical bio-loggers without this complication [47]. Potential breaches in surgical sterility during field procedures might have increased the risk of bacterial engraftment on biomaterials, potentially leading to increased susceptibility to infections [48]. Infections and delayed healing in the surgical wounds might have resulted in the rejection of subcutaneous heart rate loggers through the incision wound. This idea does not hold for the three beavers that still had the heart rate logger at the first recapture and where logger loss was documented at a subsequent capture, as normal incision healing should occur within two weeks. Finally, intraspecific aggression and wounding other beavers would be a possible mechanism [25, 49], where trauma and/or infected surgical wounds resulted in subcutaneous bio-logger loss. This latter mechanism might explain the loss of subcutaneous heart rate loggers that, in at least 3 instances, were lost after the wound was healed, because we managed to successfully download data ca. 2 months after surgery, and the individuals then lost the heart rate logger later.

For intra-abdominal temperature loggers, the loss is harder to explain. One speculation is that the temperature loggers underwent a foreign body reaction in close proximity to the gastro-intestinal tract, and were first adhered to, then engulfed by and then ejected into the gastro-intestinal tract. This has been observed in sheep (*Ovis aries*) with implanted peritoneal loggers [11]. Similarly, externalization of transmitters implanted in the peritoneal cavity was reported for channel catfish (*Ictalurus punctatus*), where transmitters had been expelled in more than half of the implanted fish within 23 days post-implantation, either through the intestine, through the incision, or (in one case) through a lesion in the ventral body wall [50]. Transmitter loss was also reported for birds and reptiles [51, 52]. In contrast, a study implanting 305 brown bears with VHF transmitters reported no loss [13]. These contrasting findings indicate that species differences (physiology, immune system etc.) might play a role regarding device loss. Moreover, implantation procedures, the coating material of implants, and suture material may play a role in wound healing and foreign body reactions [53]. We argue that using a suture type with a longer holding power (such as Maxon, or PDS, as used here) is appropriate due to the *Linea alba*’s longer time for healing than other tissue types (18–21 days). The prolene sutures, used to tack the logger to the body wall, could be avoided in future studies, either allowing the temperature loggers to be free-floating or by using a faster absorbing suture. There are species differences in reactivity to foreign bodies. Although beavers are poorly studied in this respect, studies in laboratory rodents found that Maxon and PDS were less reactive than some alternatives, including Vicryl (commonly used in veterinary medicine) [46]. The sutures monocryl and biosin were less reactive than PDS. As these sutures are absorbed in 90–120 days (vs 180–210 days for PDS), they would be a good alternative for skin sutures (for subcutaneous device closure and for closure of the skin layer after abdominal closure with suture such as PDS). Another factor is the thickness of the suture, because tissue reactivity increases with increasing suture size [54], and larger suture sizes decrease the amount of bacteria needed for a wound to get infected [55]. Experience from other species indicates that smaller sutures (2–0 PDS for *Linea alba* closure and 3–0 to 4–0 for skin closure) would most likely be sufficient.

### Conclusions and future perspectives

In conclusion, we can confirm that PIT tags are generally reliable for tagging wild animals that are hard to distinguish under field conditions. As PIT tag loss (either due to incorrect implantation or rejection by the beaver’s immune system) or failure (malfunctioning of the tag) might occasionally occur, we advocate for a combination of PIT tags with other indirect or direct methods, such as ear tagging or the recording of individual marks (e.g. tail scars in the case of beavers). Only the combination with ear tags (that are lost more frequently than PIT tags) and recording of tail scars (that change over time) allowed us to reliably identify > 450 individuals over > 20 years of the Norwegian beaver project. To minimize possible PIT tag ejection, the puncture wound could be sealed using tissue adhesive [56]. Incorrect implantation technique was likely the main cause for PIT tag loss, making adequate training of staff a priority to improve tag retention.

The high proportion of temperature and heart rate logger loss has animal welfare implications, because many study animals underwent surgery without subsequently providing information, consequently increasing the number of study animals needed to obtain statistically sufficient data. Thus, future studies should aim to increase both the recapture rate as well as develop mechanisms that ensure improved implant retention. Existing data and literature on implant function, reliability, and complications should be evaluated for any species where researchers plan to use surgical implants. Moreover, we recommend that future studies in new species initially test devices and procedures in a controlled environment (e.g. animals kept in captivity) to ensure post-surgery monitoring and secure data collection, including testing of different suture materials for both efficacy and reactivity in that particular species. Finally, future studies should investigate if and how the coating material of implants affects foreign body reactions. To that end, bacterial cultures and histopathology of the tissues associated with foreign body reactions to loggers and sutures could add valuable information to improve the understanding of mechanisms responsible for bio-logger retention and loss.

## Supplementary Information

**Additional file 1: Table S1.** Overview of all body temperature loggers implanted to Eurasian beavers (*Castor fiber*), showing the beaver ID, sex, temperature logger model, and fate of the logger. **Table S2.** Overview of all heart rate loggers implanted to Eurasian beavers (*Castor fiber*), showing the beaver ID, sex, heart rate logger model, and fate of the logger. **Table S3.** Estimate, standard error (SE), lower (LCI) and upper (UCI) 95% confidence interval, and *P*-values of explanatory variables for the full model analyzing the probability of PIT tag loss in a given year. Female sex and the age class ‘>3 year old’ were used as reference level. Note that the intercept only model was the highest-ranking model based on AIC. **Table S4.** Estimate, standard error (SE), lower (LCI) and upper 95% confidence interval (UCI), and *P*-values of explanatory variables for the full model analyzing the probability of (1) temperature logger loss and (2) heart rate logger loss. Female sex, temperature logger type ‘Centi-T’, and heart rate logger type ‘RVL-LINQ‘ were used as reference level. Note that the intercept only model was the highest-ranking model based on AIC.

## Data Availability

All relevant data is provided in the supplementary material (Table S1 and Table S2).
