# Supplementary material for: Retention and loss of PIT tags and surgically implanted devices in the Eurasian beaver
Source: BMC Vet Res. 2022 Jun 10;18:219. doi: 10.1186/s12917-022-03333-1 (PMC9188177; doi:10.1186/s12917-022-03333-1)
Supplement: Supplementary file 1 — Additional file 1: Table S1. Overview of all body temperature loggers implanted to Eurasian beavers (Castor fiber), showing the beaver ID, sex, temperature logger model, and fate of the logger. Table S2. Overview of all heart rate loggers implanted to Eurasian beavers (Castor fiber), showing the beaver ID, sex, heart rate logger model, and fate of the logger. Table S3. Estimate, standard error (SE), lower (LCI) and upper (UCI) 95% confidence interval, and P-values of explanatory variables for the full model analyzing the probability of PIT tag loss in a given year. Female sex and the age class ‘>3 year old’ were used as reference level. Note that the intercept only model was the highest-ranking model based on AIC. Table S4. Estimate, standard error (SE), lower (LCI) and upper 95% confidence interval (UCI), and P-values of explanatory variables for the full model analyzing the probability of (1) temperature logger loss and (2) heart rate logger loss. Female sex, temperature logger type ‘Centi-T’, and heart rate logger type ‘RVL-LINQ‘ were used as reference level. Note that the intercept only model was the highest-ranking model based on AIC. [file 12917_2022_3333_MOESM1_ESM.docx]

**Supplementary material**

Table S1: Overview of all body temperature loggers implanted to Eurasian beavers (*Castor fiber*), showing the beaver ID, sex, temperature logger model, and fate of the logger.

| Beaver ID | Sex | Model | Date implanted | Fate |
| --- | --- | --- | --- | --- |
| Anders | Male | Centi-T | 11.10.2016 | Recovered |
| Anders | Male | Micro-T | 17.08.2015 | Lost, confirmed by x-ray |
| Apple | Female | Centi-t | 11.10.2016 | Lost, confirmed by x-ray |
| Apple | Female | Micro-T | 18.08.2015 | Recovered |
| Asun | Female | Micro-T | 20.08.2015 | Not recaptured |
| Athena | Female | Centi-T | 18.08.2015 | Recovered |
| Betty | Female | Centi-T | 03.05.2017 | Not recaptured |
| Caesar | Male | Centi-T | 18.08.2015 | Recovered |
| Caesar | Male | Centi-T | 11.10.2016 | Recovered |
| Darwin | Male | Centi-T | 20.10.2015 | Recovered using x-ray |
| Dylan | Male | Micro-T | 19.08.2015 | Lost, confirmed by x-ray |
| Guri | Female | Centi-T | 04.10.2017 | Not recaptured |
| Gustav | Male | Centi-T | 03.05.2017 | Not recaptured |
| Ivo | Male | Centi-T | 11.10.2016 | Not recaptured |
| Ivo | Male | Micro-T | 19.08.2015 | Recovered |
| Kari | Female | Centi-T | 03.10.2017 | Recovered using x-ray |
| Kim | Male | Centi-T | 04.10.2017 | Recovered using x-ray |
| Kristoffer | Male | Centi-T | 04.10.2017 | Not recaptured |
| Lasse | Male | Centi-T | 21.08.2015 | Lost, confirmed by x-ray |
| Live | Female | Centi-T | 17.08.2015 | Not recaptured |
| Malena | Female | Centi-T | 21.10.2015 | Lost, confirmed by x-ray |
| Malena | Female | Centi-T | 12.10.2016 | Recovered using x-ray |
| Morten | Male | Centi-T | 10.10.2016 | Not found, likely lost |
| Morten | Male | Centi-T | 18.08.2015 | Recovered |
| Moses | Male | Centi-T | 18.08.2018 | Not recaptured |
| Paddy | Male | Micro-T | 21.08.2015 | Not recaptured |
| Pam | Female | Micro-T | 21.08.2015 | Recovered |
| Roger | Male | Centi-T | 02.05.2017 | Not recaptured |
| Rory | Male | Centi-T | 22.10.2015 | Not found, likely lost |
| Tanja | Female | Centi-T | 12.10.2016 | Recovered using x-ray |
| Tanja | Female | Micro-T | 19.08.2015 | Lost, confirmed by x-ray |
| Thomas | Male | Centi-T | 11.10.2016 | Not recaptured |
| Thomas | Male | Micro-T | 19.08.2015 | Not found, likely lost |
| Victoria | Female | Micro-T | 19.08.2015 | Not recaptured |
| Vigdis | Female | Centi-T | 03.10.2017 | Not recaptured |
| Waltraut | Female | Centi-T | 20.08.2015 | Recovered |

Table S2: Overview of all heart rate loggers implanted to Eurasian beavers (*Castor fiber*), showing the beaver ID, sex, heart rate logger model, and fate of the logger.

| Beaver ID | Sex | Model | Date implanted | Fate |
| --- | --- | --- | --- | --- |
| Anders | Male | RVL-BW | 11.10.2016 | Lost |
| Apple | Female | RVL-BW | 11.10.2016 | Lost |
| Athena | Female | RVL-BW | 18.08.2015 | Recovered |
| Athena | Female | RVL-XT | 05.11.2015 | Recovered |
| Caesar | Male | RVL-BW | 11.10.2016 | Lost |
| Darwin | Male | RVL-BW | 20.10.2015 | Data downloaded once, then lost |
| Gustav | Male | Linq | 03.05.2017 | Data downloaded once; not recaptured afterwards |
| Ivo | Male | RVL-BW | 11.10.2016 | Not recaptured |
| Kari | Female | Linq | 03.10.2017 | Lost |
| Kristoffer | Male | Linq | 04.10.2017 | Not recaptured |
| Lasse | Male | RVL-BW | 21.08.2015 | Data downloaded once, then lost |
| Live | Female | RVL-BW | 17.08.2015 | Lost |
| Malena | Female | RVL-BW | 06.11.2015 | Lost |
| Malena | Female | RVL-XT | 21.10.2015 | Recovered |
| Morten | Male | RVL-BW | 18.08.2015 | Recovered |
| Morten | Male | RVL-XT | 30.11.2015 | Recovered |
| Moses | Male | RVL-BW | 18.08.2018 | Not recaptured |
| Roger | Male | Linq | 02.05.2017 | Recovered |
| Rory | Male | RVL-BW | 22.10.2015 | Data downloaded once, then lost |
| Tanja | Female | RVL-BW | 12.10.2016 | Lost |
| Waltraut | Female | RVL-BW | 20.08.2015 | Recovered |

Table S3: Estimate, standard error (SE), lower (LCI) and upper (UCI) 95% confidence interval, and *P*-values of explanatory variables for the full model analyzing the probability of PIT tag loss in a given year. Female sex and the age class ‘>3 year old’ were used as reference level. Note that the intercept only model was the highest-ranking model based on AIC.

| Variable | Estimate | SE | LCI | UCI | *P*-value |
| --- | --- | --- | --- | --- | --- |
| Intercept | -9.73 | 2.09 | -13.82 | -5.63 | <0.001 |
| Sex male | 1.08 | 2.12 | -3.07 | 5.23 | 0.61 |
| Age class 2-3 year old | 0.26 | 0.70 | -1.12 | 1.64 | 0.71 |
| Age class yearling | 1.10 | 0.93 | -0.73 | 2.93 | 0.24 |

Table S4: Estimate, standard error (SE), lower (LCI) and upper 95% confidence interval (UCI), and *P*-values of explanatory variables for the full model analyzing the probability of (1) temperature logger loss and (2) heart rate logger loss. Female sex, temperature logger type ‘Centi-T’, and heart rate logger type ‘RVL-LINQ‘ were used as reference level. Note that the intercept only model was the highest-ranking model based on AIC.

| Variable | Estimate | SE | LCI | UCI | P-value |
| --- | --- | --- | --- | --- | --- |
| (1) Temperature loggers |  |  |  |  |  |
| Intercept | -1.22 | 0.80 | -3.03 | 0.22 | 0.125 |
| Sex male | 0.73 | 0.92 | -1.03 | 2.66 | 0.426 |
| Sensor type Micro-T | 1.10 | 0.95 | -0.74 | 3.09 | 0.247 |
|  |  |  |  |  |  |
| (2) Heart rate loggers |  |  |  |  |  |
| Intercept | -0.78 | 1.39 | -4.09 | 1.89 | 0.58 |
| Sex male | 0.13 | 0.98 | -1.81 | 2.13 | 0.90 |
| Sensor type RVL-DX/XT | 1.13 | 1.35 | -1.45 | 4.35 | 0.41 |
